# Ameliorative Effects of Soybean Powder Fermented by *Bacillus subtilis* on Constipation Induced by Loperamide in Rats

**DOI:** 10.3390/ijms26157615

**Published:** 2025-08-06

**Authors:** Gi Soo Lee, Su Kang Kim, Ju Yeon Ban, Chung-Hun Oh

**Affiliations:** 1 Department of Oral Physiology, College of Dentistry, Dankook University, Cheonan 31116, Republic of Korea; dlrltn167@gmail.com; 2 Department of Medical Laser, Graduate School, Dankook University, Cheonan 31116, Republic of Korea; 3 Department of Biomedical Laboratory Science, Catholic Kwandong University, Gangneung 25601, Republic of Korea; skkim7@cku.ac.kr; 4 Department of Dental Pharmacology, College of Dentistry, Dankook University, Cheonan 31116, Republic of Korea

**Keywords:** *Bacillus subtilis*, fermented soybean, constipation, probiotics, ioflavones, intestinal motility, loperamide model

## Abstract

Constipation is a prevalent gastrointestinal disorder that significantly impairs quality of life. While pharmacological agents such as loperamide are widely used to induce constipation in experimental models, there is increasing interest in natural alternatives for alleviating intestinal dysfunction. In this study, we investigated the laxative effects of soybean powder fermented by *Bacillus subtilis* DKU_09 in a loperamide-induced rat model of constipation. The probiotic strain was isolated from cheonggukjang, a traditional Korean fermented soybean paste, and its identity was confirmed through 16S rRNA sequencing. Fermented soybean powder was characterized morphologically via scanning electron microscopy and chemically via HPLC to assess its isoflavone content. Rats were administered loperamide (5 mg/kg) for four days to induce constipation and were then treated with fermented soybean powder at doses of 100, 200, or 300 mg/kg. No pharmacological laxatives (e.g., PEG) were used as a positive control; instead, values from the treatment groups were compared with those from the loperamide-only constipation group. Key outcomes of fecal output, water content, colonic fecal retention, and gastrointestinal transit ratio were measured. The fermented product significantly improved stool frequency and moisture content, reduced colonic fecal retention, and restored gastrointestinal transit in a dose-dependent manner. Notably, the 300 mg/kg group demonstrated nearly complete recovery of fecal parameters without affecting body weight. Statistical analysis was performed using one-way ANOVA followed by Tukey’s post hoc test. These findings suggest that *Bacillus subtilis*-fermented soybean powder exerts synergistic laxative effects through the combined action of probiotic viability and fermentation-enhanced bioactive compounds such as aglycone isoflavones. This study supports the potential use of fermented soybean-based nutraceuticals as a natural and safe intervention for constipation and gastrointestinal dysregulation.

## 1. Introduction

Constipation is a prevalent gastrointestinal disorder characterized by infrequent defecation, hard stools, and a sensation of incomplete evacuation. The condition is associated with considerable discomfort and a significant impairment in quality of life, often leading to secondary complications such as abdominal pain, nausea, vomiting, abdominal distension, anorexia, and headache [1].

Although various pharmacological treatments are available, their long-term use is frequently limited by adverse effects and the emergence of drug tolerance [2,3]. Polyethylene glycol (PEG), for instance, is a frequently utilized laxative agent; however, its prolonged administration can result in a variety of adverse effects, including abdominal bloating, cramps, and a reduction in therapeutic efficacy.

Consequently, there is a growing interest in dietary strategies and functional food-based therapies, which offer favorable safety profiles and are suitable for long-term use [4,5]. Among various dietary components, fermented foods have attracted increasing scientific attention due to their ability to modulate the composition and activity of the gut microbiota and to improve gastrointestinal function. In particular, fermented foods containing probiotic strains such as *Bacillus subtilis* or *Lactobacillus* spp. have been shown to enhance the production of microbiota-derived bioactive compounds, including short-chain fatty acids (SCFAs; e.g., acetate, propionate, and butyrate), as well as digestive enzymes such as amylase, protease, and lipase [6,7].

*Bacillus subtilis* is a spore-forming, non-pathogenic Gram-positive bacterium that is widely utilized as a probiotic owing to its ability to withstand harsh gastrointestinal conditions and confer multiple health benefits [8]. The spores of this organism are resistant to heat and acidity, enabling survival through the stomach and germination in the intestine, where they produce various enzymes (e.g., proteases, amylases, lipases), vitamins (e.g., K_2_, B-complex), and antimicrobial compounds such as bacteriocins [9,10].

The utilization of dietary supplementation with *Bacillus subtilis* has been reported to exert a positive influence on the integrity of the intestinal barrier, promote the optimal morphology of the intestinal tract, and augment the proliferation of beneficial microbial populations, including *Akkermansia muciniphila* [9]. Clinically, a 6-week randomized, double-blind trial demonstrated that *Bacillus subtilis* BS50 supplementation significantly alleviated gastrointestinal symptoms, such as bloating and gas, and increased stool frequency and consistency [9]. In addition to these gastrointestinal effects, the health benefits of *Bacillus subtilis* are partly mediated by its exopolysaccharides, which exhibit anti-inflammatory activity by promoting M2 macrophage differentiation and protecting against intestinal inflammation in murine models [11].

Soybeans are a rich source of isoflavones and bioactive peptides, and their fermentation by *Bacillus strains* significantly enhances their functional properties. The process has been shown to increase the concentration of aglycone isoflavones, such as daidzein and genistein, which have been found to be more readily absorbed and exhibit stronger antioxidant and anti-inflammatory activity than their glycoside forms [12,13]. Furthermore, the process of fermentation has been demonstrated to yield antioxidant peptides and phenolic compounds, which have been shown to enhance radical scavenging capacity [12]. Fermented soy products have been shown to inhibit the expression of inflammatory genes, such as COX-2 and iNOS, and to decrease cytokine levels, including TNF-α and IL-6, in both cellular and animal models [14,15]. Taken together, these findings highlight the role of fermented soy products in maintaining intestinal barrier function and shaping a beneficial gut microbial environment, thereby underpinning their anti-inflammatory and gastrointestinal health-promoting effects.

Traditionally, fermented soybean foods have been used as natural remedies for constipation. Recent animal studies confirm that soybean fermented with *Bacillus subtilis* significantly improves fecal output, increases stool moisture, and accelerates colonic transit in loperamide-induced constipation models [16]. These laxative effects are thought to be mediated by both microbial metabolites and mucilaginous polysaccharides produced during fermentation, which contribute to improved intestinal motility and stool hydration [16].

Given these findings, we hypothesized that soybean powder fermented by *Bacillus subtilis* may exert beneficial effects in alleviating constipation. In this study, we investigated the laxative effects of *Bacillus subtilis*-fermented soybean powder using a loperamide-induced rat model of constipation. The therapeutic effects were evaluated by analyzing fecal parameters, gastrointestinal transit, and stool water content. Our findings aim to provide scientific evidence supporting the application of naturally fermented foods in promoting gastrointestinal health.

## 2. Results

### 2.1. Morphological and Chemical Characteristics of Bacillus subtilis-Fermented Soybean Powder

When a single colony of the *Bacillus subtilis* DKU_09 strain was sequenced for the 16S rRNA gene, BLAST-based phylogenetic analysis showed a 99.91% similarity to *Bacillus subtilis* for 1111 base sequences. *Bacillus subtillis* has been designated as a harmless strain by the Ministry of Food and Drug Safety of the Republic of Korea (KFDA) when ingested, similar to strains such as *Bacillus coagulans*, *Bacillus natto*, and *Bacillus polyfermenticus*, and is known to not exhibit toxicity when administered to experimental animals such as rats, rabbits, guinea pigs, and pigs [17,18]. Consequently, it is deemed appropriate for utilization as a probiotic and was employed for soybean fermentation in the present study.

**Figure 1:**
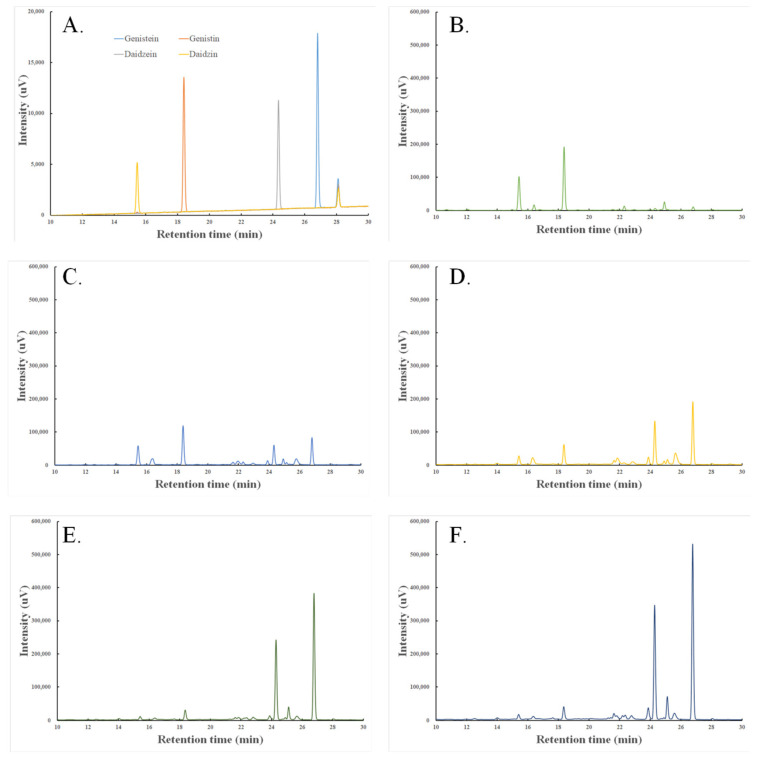
HPLC profiles and isoflavone composition of soybean fermented by *Bacillus subtilis* DKU_09. (**A**) Standard, (**B**) non fermented soybean, (**C**) 1 day fermented soybean, (**D**) 2 day fermented soybean, (**E**) 3 day fermented soybean, (**F**) 4 day fermented soybean. The retention times (RT) of the identified standard compounds were: daidzin ≈ 15.0 min, genistin ≈ 18.0 min, daidzein ≈ 24.5 min, and genistein ≈ 26.5 min. The concentration of each compound was quantified based on peak areas and expressed in μg/g.

High-performance liquid chromatography (HPLC) analysis revealed distinct isoflavone profiles in soybeans fermented by *Bacillus subtilis* DKU_09. Figure 1 and Table 1 present representative HPLC chromatograms illustrating the compositional changes of major soy isoflavones—daidzein, daidzin, genistein, and genistin—during fermentation (Day 0 to Day 4). Over the course of fermentation, a gradual decline in the peaks associated with the glycoside forms (daidzin and genistin) was observed, accompanied by a corresponding increase in the aglycone forms (daidzein and genistein). These changes are indicative of enzymatic hydrolysis processes, whereby isoflavone glycosides are converted into their aglycone counterparts by microbial activity. In addition to the major isoflavone peaks, several minor, unidentified peaks were also detected in the chromatograms. These peaks may correspond to intermediate fermentation metabolites or minor bioactive compounds generated during enzymatic hydrolysis, and their characterization will require further analysis (e.g., LC-MS/MS) in future studies.

**Figure 2:**
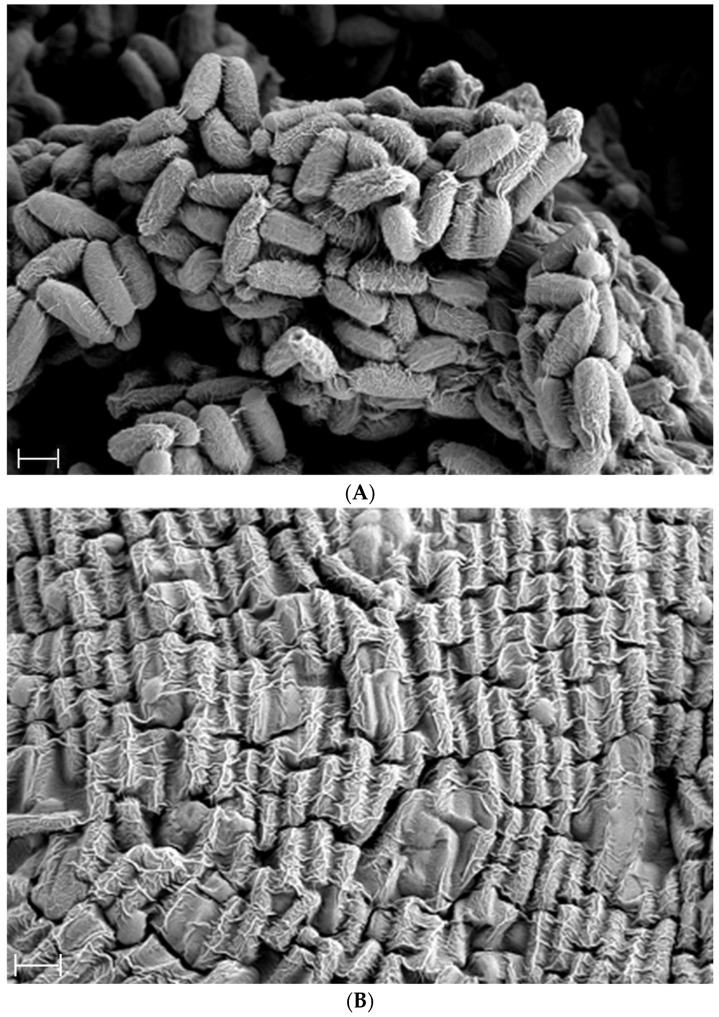
Scanning electron microscopy (SEM) images of *Bacillus subtilis* DKU_09 showing typical rod-shaped morphology with visible endospores (**A**) and vegetative cells (**B**). Images were captured at 15,000× magnification using SE2 detection. Scale bars = 1 µm.

The SEM confirmed the presence of endospores and vegetative cells, with typical rod-shaped morphology and dense clustering, suggesting active proliferation and viability of the probiotic strain (Figure 2).

### 2.2. Body Weight Changes in Loperamide-Induced Constipated Rats

**Figure 3:**
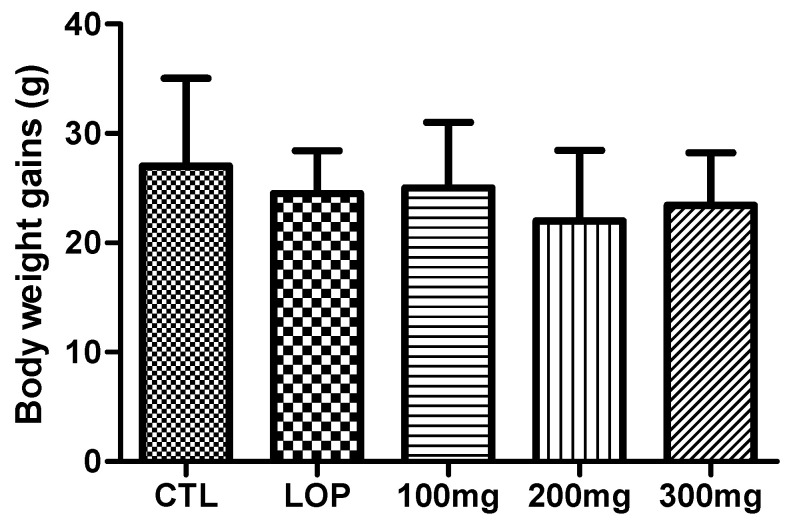
Effects of loperamide and *Bacillus subtilis* DKU_09-fermented soybean powder on rat body weight gains. Body weights of rats were measured every morning at 9 a.m. and the weight difference between the time of inducing constipation and the time of sacrifice was calculated and expressed as grams (mean ± SD; *n* = 5). CTL: not loperamide-induced and not treated with fermented soybean powder; LOP: loperamide-induced and not treated with fermented soybean powder; 100 mg: loperamide-induced and treated with fermented soybean powder 100 mg/kg; 200 mg: loperamide-induced and treated with fermented soybean powder 200 mg/kg; 300 mg: loperamide-induced and treated with fermented soybean powder 300 mg/kg.

Animals were weighed on the first day of loperamide treatment and before sacrifice to determine the amount of body weight change during the study to determine if the administration of loperamide and fermented soybean powder affected body weight gain. The change in body weight in the loperamide alone group was not different from that of the non-loperamide group, nor was it significantly different from that of the fermented soybean powder groups (Figure 3). The results of this study are similar to those of a previous study that showed that loperamide had no significant effect on body weight change. This suggests that the loperamide and fermented soybean powder administered to rats in this study were nontoxic.

### 2.3. Effects on Fecal Pellet Output

**Figure 4:**
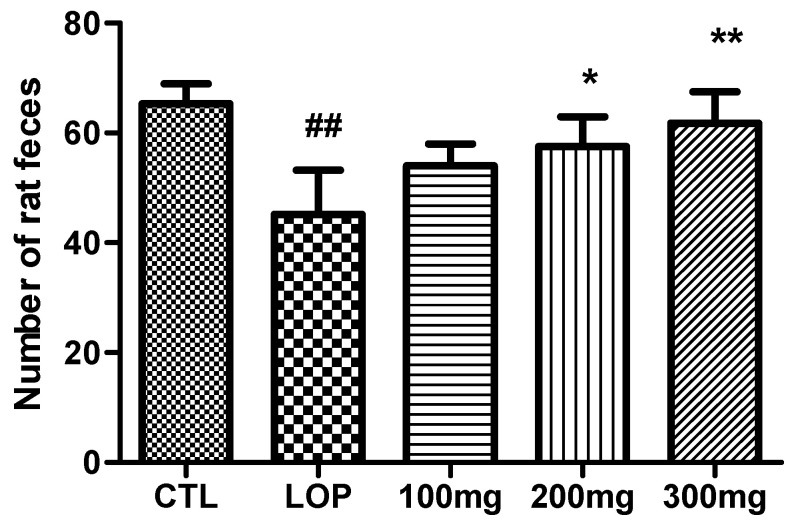
Number of fecal pellets in loperamide-induced constipated rats. Fecal pellets were collected for 24 h on the 3rd day after loperamide induction and expressed as number of rat feces (mean ± SD; *n* = 5). CTL: not loperamide-induced and not treated with fermented soybean powder; LOP: loperamide-induced and not treated with fermented soybean powder; 100 mg: loperamide-induced and treated with fermented soybean powder 100 mg/kg; 200 mg: loperamide-induced and treated with fermented soybean powder 200 mg/kg; 300 mg: loperamide-induced and treated with fermented soybean powder 300 mg/kg. ^##^ *p* < 0.001 vs. CTL group; * *p* < 0.05, ** *p* < 0.001 vs. LOP group by Tukey’s multiple comparison test.

To ascertain whether constipation was induced in the experimental animals, the number of fecal pellets in the CTL and LOP groups was compared. When loperamide was administered, the number of fecal pellets decreased from an average of 65.3 ± 3.6 in the non-loperamide group to 45.2 ± 8.1, supporting that loperamide administration was effective in inducing constipation (*p* < 0.001). The number of fecal pellets in the groups orally administered 100 mg/kg, 200 mg/kg, and 300 mg/kg body weight of fermented soybean powder increased in a dose-dependent manner, with an average of 54.0 ± 4.0, 57.5 ± 5.4, and 61.8 ± 5.7, respectively, compared to the loperamide-only treatment group (Figure 4).

### 2.4. Effects on Fecal Water Content

**Figure 5:**
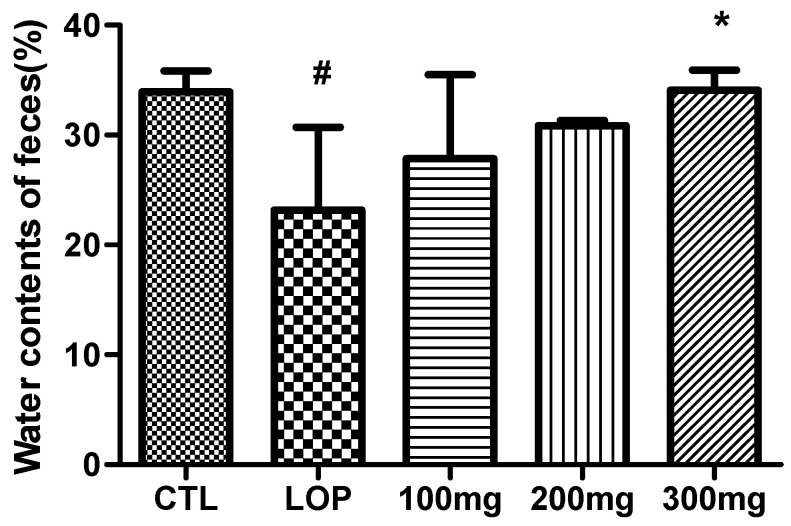
Water contents of fecal pellets in loperamide-induced constipated rats. Fecal pellets were collected for 24 h on the 3rd day after loperamide induction and weighed before and after drying for 48 h at 70 °C. Water contents of feces were calculated as the difference between wet and dried weights of feces and expressed as percentage (mean ± SD; *n* = 5). CTL: not loperamide-induced and not treated with fermented soybean powder; LOP: loperamide-induced and not treated with fermented soybean powder; 100 mg: loperamide-induced and treated with fermented soybean powder 100 mg/kg; 200 mg: loperamide-induced and treated with fermented soybean powder 200 mg/kg; 300 mg: loperamide-induced and treated with fermented soybean powder 300 mg/kg. ^#^ *p* < 0.05 vs. CTL group; * *p* < 0.05 vs. LOP group by Tukey’s multiple comparison test.

The fecal water content in the CTL without loperamide administration was 33.9 ± 1.9%. In contrast, loperamide treatment significantly reduced the fecal water content to 23.2 ± 7.5%. However, administration of fermented soybean powder dose-dependently increased the fecal water content, with the 300 mg/kg group showing partial recovery to 34.1 ± 1.8%, a level comparable to the CTL group (Figure 5).

Given that one of the hallmark symptoms of constipation is the reduction of stool water content and the inhibition of intestinal water secretion [19], these results confirm that loperamide successfully induced constipation, as evidenced by reduced fecal moisture. Furthermore, the administration of fermented soybean powder effectively alleviated this symptom by restoring the fecal water content.

### 2.5. Colonic Fecal Retention

**Figure 6:**
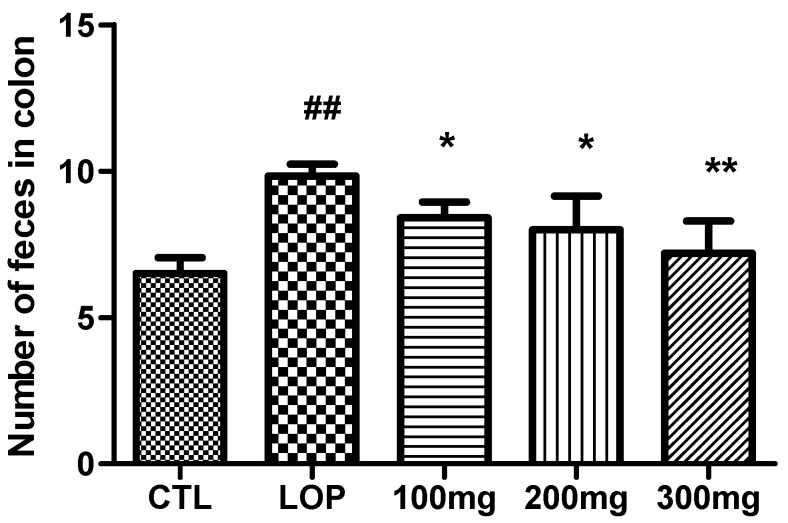
Number of fecal pellets in colon in loperamide-induced constipated rats. Fecal pellets in distal colon were counted after sacrifice of rats and expressed as number of feces in colon (mean ± SD; *n* = 5). CTL: not loperamide-induced and not treated with fermented soybean powder; LOP: loperamide-induced and not treated with fermented soybean powder; 100 mg: loperamide-induced and treated with fermented soybean powder 100 mg/kg; 200 mg: loperamide-induced and treated with fermented soybean powder 200 mg/kg; 300 mg: loperamide-induced and treated with fermented soybean powder 300 mg/kg. ^##^ *p* < 0.001 vs. CTL group; * *p* < 0.05, ** *p* < 0.001 vs. LOP group by Tukey’s multiple comparison test.

The number of residual feces in the colon in the CTL group was 6.5 ± 0.5, and in the group administered loperamide (LOP group), the number of residual feces in the colon was 9.8 ± 0.4, which was significantly increased compared to the CTL group. When 100 mg/kg, 200 mg/kg, and 300 mg/kg body weight of fermented soybean powder was administered, the number of residual feces in the colon decreased to 8.4 ± 0.5, 8.0 ± 1.2, and 7.2 ± 1.1, respectively (Figure 6). According to previous studies, it is known that when constipation is induced by loperamide administration, the number of feces in the colon increases [20], and in this study, the number of residual feces in the colon of rats administered loperamide increased, showing similar results, and it was confirmed that the number of residual feces decreased when fermented soybean powder was administered.

### 2.6. Improvement in Gastrointestinal Transit

**Figure 7:**
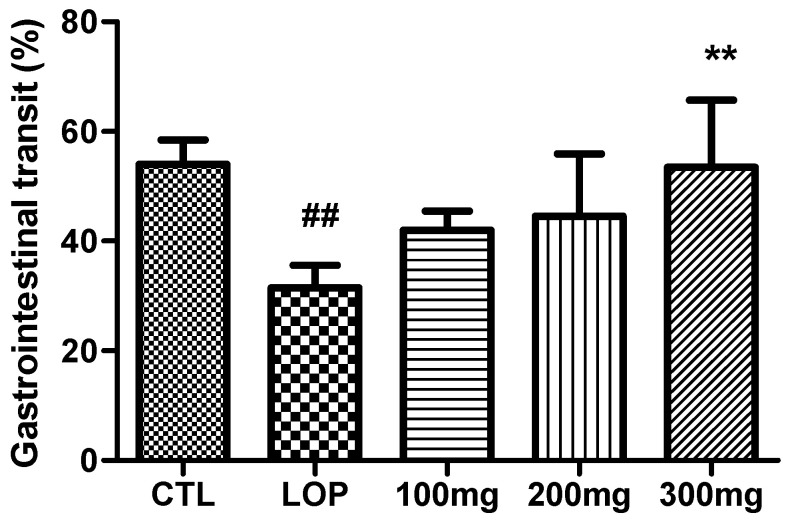
Effects of soybean fermented by *Bacillus subtilis* DKU_09 on gastrointestinal transit ratio in colon of loperamide-induced constipated rats. The distance the carmine had travelled and the total length of the colon were measured 3 h after oral administration of carmine and expressed as gastrointestinal transit ratio (mean ± SD; *n* = 5). CTL: not loperamide-induced and not treated with fermented soybean powder; LOP: loperamide-induced and not treated with fermented soybean powder; 100 mg: loperamide-induced and treated with fermented soybean powder 100 mg/kg; 200 mg: loperamide-induced and treated with fermented soybean powder 200 mg/kg; 300 mg: loperamide-induced and treated with fermented soybean powder 300 mg/kg. ^##^ *p* < 0.001 vs. CTL group; ** *p* < 0.001 vs. LOP group by Tukey’s multiple comparison test.

The gastrointestinal transit rate of feces was measured by calculating the ratio of the distance carmine passed through the gastrointestinal tract to the length of the gastrointestinal tract. The gastrointestinal transit rate of the CTL group was 54.0 ± 4.0%, and it decreased to 31.5 ± 4.1% when loperamide was administered alone. Loperamide is known to activate µ-opioid receptors and decrease intestinal motility [21,22]. Based on this, in this study, it was confirmed that loperamide administration decreases the gastrointestinal transit rate of carmine, thereby decreasing intestinal motility and ultimately causing bowel movement disorders and increasing the number of residual feces in the intestine (Figure 7). In addition, when compared to the previous results showing that the administration of *B. subtilis* partially improved the intestinal motility reduced by loperamide [23], in this study, when loperamide and fermented soybean powder were administered together at 100 mg/kg, 200 mg/kg, and 300 mg/kg body weight, the gastrointestinal transit rates were 42.0 ± 3.5%, 44.5 ± 11.4%, and 53.4 ± 12.3%, respectively, indicating the fermented soybean powder increased intestinal motility compared to loperamide alone, which suggests a potential association with improved intestinal transit and stool output.

## 3. Discussion

In this study, we demonstrated that soybeans fermented by *Bacillus subtilis* DKU_09 exhibited significant laxative effects in a loperamide-induced rat model of constipation. The treatment notably increased fecal output and moisture content, reduced fecal retention in the colon, and improved gastrointestinal transit, indicating enhanced intestinal motility and stool hydration.

Loperamide is widely used to induce constipation in animal models by inhibiting gastrointestinal motility and fluid secretion via μ-opioid receptor activation [24]. This model effectively replicates key features of human functional constipation, including reduced stool frequency, hardened stools, and delayed intestinal transit [25]. Consistent with these characteristics, rats in the loperamide-injected group exhibited significant reductions in fecal parameters and gastrointestinal motility, which were effectively reversed by treatment with soybean powder fermented by *Bacillus subtilis* DKU_09.

Previous studies have reported several underlying mechanisms that may account for the observed laxative effects of fermented soy products. In this study, HPLC revealed a progressive increase in aglycone isoflavones, such as daidzein and genistein, alongside a concurrent decrease in their glycoside counterparts (daidzin and genistin) over the 4-day fermentation period. The retention times and peak areas of these compounds were clearly distinguishable, indicating enzymatic hydrolysis by *Bacillus subtilis* DKU_09, which converts glycosides into more bioavailable aglycones.

In addition, several unidentified peaks emerged during fermentation. While their exact chemical identities and biological roles remain unclear, these peaks may represent minor metabolites or degradation products generated through microbial fermentation. Due to the limitations of the current analytical method, we refrained from further speculation on their functional relevance.

Aglycone isoflavones are known to possess antioxidant, anti-inflammatory, and pro-motility properties [26,27]. These aglycones are more readily absorbed and bioactive compared to their glycoside precursors, thereby enhancing their physiological effects in the gut [28]. Recent studies have demonstrated that the fermentation of soy products enhances their content of bioactive peptides and phenolic compounds, which exert anti-inflammatory effects by downregulating the expression of pro-inflammatory cytokines such as TNF-α and IL-6, as well as inflammatory enzymes including iNOS and COX-2—molecules frequently elevated in gastrointestinal disorders [29,30].

Secondly, the presence of *Bacillus subtilis* itself likely contributed to the observed improvements. SEM images further confirmed the morphological integrity and viability of the strain, showing densely clustered rod-shaped vegetative cells and endospores. These features support the strain’s ability to actively proliferate and adapt to harsh gastrointestinal conditions, reinforcing its probiotic potential. *Bacillus subtilis* is a robust probiotic capable of surviving gastric acidity and colonizing the intestine, where it produces various enzymes and antimicrobial metabolites. These actions can promote gut microbial balance, enhance mucosal immunity, and stimulate peristalsis through SCFA production [7,31]. Furthermore, exopolysaccharides derived from *Bacillus subtilis* may help maintain intestinal barrier function and exert anti-inflammatory effects, which could collectively ameliorate constipation symptoms [11].

Notably, our data revealed a dose-dependent improvement in fecal parameters and transit ratio, with the 300 mg/kg group showing the most significant effects across all measures. This suggests that both the bioactive compounds and the probiotic content of the fermented soybean powder contribute synergistically to gastrointestinal regulation. Importantly, oral administration of the fermented product did not cause significant changes in body weight, indicating that it is physiologically safe and well-tolerated.

These findings are in line with previous studies reporting the laxative and gut-modulating effects of fermented soy products and *Bacillus subtilis* supplementation [16]. However, this study is among the first to investigate the combined effect of soybean fermentation by *Bacillus subtilis* on both stool output and gut motility in a constipated animal model.

Despite the promising results, several limitations of the present study should be acknowledged. Most notably, this study did not include a non-fermented soybean control group, which limits the ability to determine whether the observed effects are attributable specifically to fermentation-derived bioactivities or to the general nutritional matrix of soybeans. While s previous study reported beneficial outcomes using fermented soy products without non-fermented comparators [8], we recognize that the inclusion of a non-fermented soybean group would have enabled a more precise mechanistic interpretation by distinguishing between the baseline effects of soybean itself and the additional benefits conferred by fermentation. We fully acknowledge this as a critical limitation and plan to address it in future studies.

Additionally, although our study demonstrated clear functional improvements in constipation-related parameters, we did not perform biochemical analyses of fermentation-derived bioactive compounds, such as specific peptides or low-molecular-weight metabolites, which are known to play a key role in modulating gut physiology. The lack of characterization of these components limits our ability to link the observed physiological effects to specific molecular mechanisms. Given that bioactive peptides generated during fermentation are increasingly recognized for their anti-inflammatory, antioxidant, and pro-motility effects, their identification and quantification would substantially enhance the mechanistic depth and translational value of our findings.

This study was conducted using a short-term animal model, which allowed for the assessment of acute effects. However, the long-term effects of fermented soybean intake on gut health, including mucosal integrity, microbiota modulation, and immune responses, remain to be elucidated. Furthermore, key pathways such as SCFA production, mucin expression, and microbial community shifts were not assessed and warrant comprehensive investigation in subsequent research.

Finally, the dosage of fermented soybean powder was determined based on prior preclinical studies and, when converted to human-equivalent doses, falls within a range potentially suitable for dietary supplementation. Nonetheless, rigorous clinical studies will be necessary to validate its efficacy, determine its optimal dosage, and confirm its safety in human populations.

## 4. Materials and Methods

### 4.1. Isolation and Identification of Bacillus subtilis DKU_09

The *Bacillus subtilis* DKU_09 strain was isolated from cheonggukjang and cultured in Tryptic Soy Broth (TSB; BD, Franklin Lakes, NJ, USA) using a shaking incubator at 40 °C and 150 rpm for 24 h. A single colony was then selected on Tryptic Soy Agar (TSA; BD, NJ, USA). For identification, colony DNA was extracted, and 16S rRNA gene sequencing was performed using the universal primers 27F (5′-AGA GTT TGA TCM TGG CTC AG-3′), 1492R (5′-TAC GGY TAC CTT GTT ACG ACT T-3′), 785F (5′-GGA TTA GAT ACC CTG GTA-3′), and 907R (5′-CCG TCA ATT CMT TTR AGT TT-3′) by Macrogen (Seoul, Republic of Korea). Sequence homology was determined via the Basic Local Alignment Search Tool (BLAST, version 2.17.0) provided by the National Center for Biotechnology Information (NCBI, Bethesda, MD, USA) database (Table 2).

### 4.2. Preparation of Fermented Soybean and Morphological Analysis

To prepare the fermentation starter, the *Bacillus subtilis* DKU_09 strain was cultured in TSB at 42 °C with shaking at 150 rpm for 18 h. Soybeans were pretreated by soaking at 4 °C for 8 h, followed by boiling at 121 °C for 1 h. The boiled soybeans were inoculated with the *Bacillus subtilis* DKU_09 starter culture and incubated at 37 °C for 24 h. After fermentation, the soybeans were sequentially dried at 42 °C for 48 h and then at 70 °C for another 48 h. To assess viable bacteria, the fermented powder was diluted to 10^8^ colony-forming units (CFU)/mL, plated on TSA, and incubated at 37 °C for 20 h, after which CFUs were counted. Morphological characterization of the *Bacillus subtilis* DKU_09 strain, including endospore formation during fermentation, was conducted using scanning electron microscopy (SEM). The powdered samples were fixed, dehydrated, and sputter-coated with gold prior to observation under the electron microscope.

HPLC analysis was performed using a Shimadzu Co-Sense series system (Shimadzu, Kyoto, Japan) equipped with a UV detector set at 260 nm. Chromatographic separation was achieved using a C18 reversed-phase column (250 mm × 4.6 mm, 5 μm particle size; Agilent Technologies, Santa Clara, CA, USA), maintained at a column oven temperature of 40 °C. The mobile phase consisted of solvent A (0.1% acetic acid in water) and solvent B (0.1% acetic acid in methanol). A low-pressure gradient elution was applied under the following conditions: 20% solvent B at 0 min, linearly increased to 50% at 15 min, to 70% at 25 min, and returned to 20% at 30 min. The flow rate was maintained at 1.0 mL/min, and the total analysis time was 30 min. The injection volume was 10 μL.

Sample preparation involved extracting 3 g of fermented soybean powder with 27 mL of 80% methanol. The mixture was vortexed thoroughly, followed by orbital shaking for 1 h and sonication in a water bath for 30 min. The extract was then centrifuged at 12,000 rpm for 15 min, and the supernatant was filtered through a 0.45 μm PTFE syringe filter (F17-2045, Chrom Tech, Apple Valley, MN, USA). Filtered samples were stored at −20 °C until analysis.

Quantification of isoflavones, including daidzin, daidzein, genistin, and genistein, was performed using external standard calibration curves prepared with authentic standards at a concentration of 20 μg/mL. The calibration curves demonstrated excellent linearity, with correlation coefficients (r^2^) exceeding 0.99.

### 4.3. Animal Experiments Design

**Figure 8:**
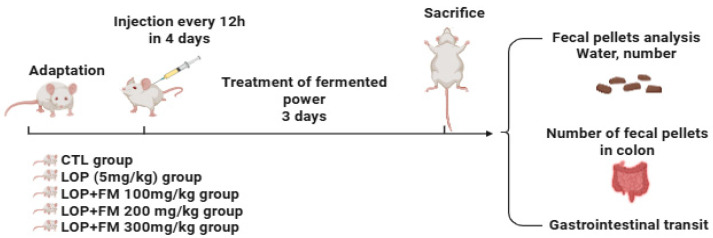
Experimental workflow for evaluating the laxative effects of fermented powder in a loperamide-induced constipation mouse model. Male mice were acclimatized and then divided into five groups: control (CTL), loperamide-treated (LOP, 5 mg/kg), and LOP co-treated with fermented powder (FM) at doses of 100, 200, or 300 mg/kg. Constipation was induced by subcutaneous injection of loperamide every 12 h for 4 consecutive days. Fermented powder was orally administered during the final 3 days of the experiment. On the final day, mice were sacrificed for evaluation. Parameters measured included total number and moisture content of fecal pellets, number of fecal pellets retained in the colon, and gastrointestinal transit using dye.

Male Sprague–Dawley (SD) rats (6 weeks old, 140–160 g) were obtained from DBL (Eumseong, Korea). Following a 1-week acclimatization period under controlled conditions (temperature: 21 ± 2 °C, humidity: 50 ± 5%), rats were housed individually with *ad libitum* access to standard chow and water. Rats were randomly assigned into five groups: control (CTL), loperamide (LOP), and fermented soybean powder treatment groups (100, 200, and 300 mg/kg body weight). Constipation was induced in the LOP and treatment groups via subcutaneous injection of loperamide (5 mg/kg, in 0.9% NaCl) every 12 h for 4 consecutive days (Figure 8) After constipation was established, fermented soybean powder was administered orally (1 mL/100 g body weight) once daily for 3 days [16]. All animal procedures were approved by the Dankook University Institutional Animal Care and Use Committee (IACUC) (DKU-23-079).

### 4.4. Total Number of Faecal Pellets, Water Contents, and Fecal Retention in the Colon

On the third day of induction of constipation, the feces in each experimental cage were removed and replaced with new bedding, then the feces were collected for 24 h. The number and weight of the feces were measured, and the weight of fecal pellets were measured again after drying in a 70° oven for 48 h. The moisture content of stools was calculated through the weight difference between before drying and after [32,33]. After sacrifice of the animals, the guts were excised from the cecum to rectum to count the number of remained feces in the colon.

### 4.5. Gastrointestinal Transit Ratio

To evaluate intestinal motility, carmine red dye (1 mL of 60 mg/mL in 0.5% carboxymethylcellulose) was orally administered 30 min after the final treatment. Three hours later, the animals were sacrificed, and the large intestine was excised. The total length of the intestine and the distance traveled by the carmine dye were measured. The gastrointestinal transit ratio was calculated to assess the effect of fermented soybean powder on intestinal motility compared to loperamide treatment.

### 4.6. Statistical Analysis

The results of this experiment are expressed as means ± SDs, and one-way analysis of variance (ANOVA) was performed using the GraphPad prism 5 program. Tukey’s multiple comparison test was utilized to determine the significance for the experimental groups and the results were considered statistically significant at *p*-values < 0.05.

## 5. Conclusions

In conclusion, while acknowledging these limitations, we believe that the present study provides foundational evidence supporting the functional benefits of *Bacillus subtilis*-fermented soybean powder in ameliorating constipation. The significant improvements in stool output, water content, colonic retention, and gastrointestinal motility observed in this short-term model suggest that fermented soy products hold promise as natural dietary interventions. To further advance the scientific basis and application of these findings, future studies should incorporate non-fermented controls, identify and characterize key fermentation-derived bioactive compounds, particularly peptides, and extend the analyses to longer-term and clinical models.

## Figures and Tables

**Table 1:** Isoflavone Content and Aglycone Ratio in Soybeans Fermented over 1–4 Days. All isoflavone values are expressed as μg/g dry weight, as determined by HPLC analysis using calibration curves from authentic standards and normalized to the dry weight of each sample.

Compound/Metric	Control	1 Day	2 Days	3 Days	4 Days
Daidzein	46.06	108.1	237.43	429.96	615.42
Daidzin	394.22	227.76	109.18	47.46	71.11
Genistein	12.52	93.9	215.37	429.17	594.96
Genistin	282.38	176.98	92.55	46.66	61.67
Total Aglycones	58.58	202	452.8	859.13	1210.39
Total Glycosides	676.6	404.74	201.73	94.12	132.78
Total Isoflavones	735.18	606.74	654.54	953.26	1343.17
Aglycone Ratio (%)	8%	33%	69%	90%	90%

**Table 2:** BLAST search for the 16S rRNA gene sequences of *Bacillus subtilis* DKU_09.

Sample	Putative Species	Related GenBank Sequence	Query Cover (%)	Identity (%)
DKU_09	*Bacillus subtilis* subsp. *subtilis*	NR_102783.2	100	99.91
	*Bacillus subtilis*	NR_116183.1	100	99.91
	*Bacillus subtilis*	NR_113265.1	100	99.91
	*Bacillus subtilis*	NR_112629.1	100	99.91
	*Bacillus subtilis*	NR_027552.1	100	99.91

## Data Availability

Data is contained within the article.
